# Supplementary material for: HIVprotI: an integrated web based platform for prediction and design of HIV proteins inhibitors
Source: J Cheminform. 2018 Mar 9;10:12. doi: 10.1186/s13321-018-0266-y (PMC5845081; doi:10.1186/s13321-018-0266-y)
Supplement: Supplementary file 2 — Additional file 2. Source code of HIVProtI web server. [file 13321_2018_266_MOESM2_ESM.zip › HIVprotI_Source-code/hivprotI/weka-3-6-6/doc/weka/core/Check.html]

Check


---


|  |  |  |
| --- | --- | --- |
| **Overview** \| **Package** \| **Class** \| **Tree** \| **Deprecated** \| **Index** \| **Help** \| **Weka's home** | |  |
| **PREV CLASS**   **NEXT CLASS** | **FRAMES**    **NO FRAMES**     **All Classes** |  |
| SUMMARY: NESTED \| FIELD \| CONSTR \| METHOD | DETAIL: FIELD \| CONSTR \| METHOD |  |


---


## weka.core Class Check

```
java.lang.Object
  weka.core.Check
```

**All Implemented Interfaces:**: OptionHandler, RevisionHandler

**Direct Known Subclasses:**: CheckGOE, CheckOptionHandler, CheckScheme

---

``` public abstract class Check extends java.lang.Object implements OptionHandler, RevisionHandler ```

Abstract general class for testing in Weka.

**Version:**
:   $Revision: 1.2 $

**Author:**
:   FracPete (fracpete at waikato dot ac dot nz)

---

**Constructor Summary**

`Check()`


| **Method Summary** | |
| --- | --- |
| `abstract  void` | `doTests()`             Begin the tests, reporting results to System.out |
| `boolean` | `getDebug()`             Get whether debugging is turned on |
| `java.lang.String[]` | `getOptions()`             Gets the current settings of the CheckClassifier. |
| `boolean` | `getSilent()`             Get whether silent mode is turned on |
| `java.util.Enumeration` | `listOptions()`             Returns an enumeration describing the available options. |
| `void` | `setDebug(boolean debug)`             Set debugging mode |
| `void` | `setOptions(java.lang.String[] options)`             Parses a given list of options. |
| `void` | `setSilent(boolean value)`             Set slient mode, i.e., no output at all to stdout |

**Methods inherited from class java.lang.Object**

`equals, getClass, hashCode, notify, notifyAll, toString, wait, wait, wait`

**Methods inherited from interface weka.core.RevisionHandler**

`getRevision`

**Constructor Detail**

### Check

```
public Check()
```


**Method Detail**

### listOptions

```
public java.util.Enumeration listOptions()
```

:   Returns an enumeration describing the available options.

    :   **Specified by:**: `listOptions` in interface `OptionHandler`
    :   **Returns:**: an enumeration of all the available options.

---


### setOptions

```
public void setOptions(java.lang.String[] options)
                throws java.lang.Exception
```

:   Parses a given list of options.

    :   **Specified by:**: `setOptions` in interface `OptionHandler`
    :   **Parameters:**: `options` - the list of options as an array of strings **Throws:**: `java.lang.Exception` - if an option is not supported

---


### getOptions

```
public java.lang.String[] getOptions()
```

:   Gets the current settings of the CheckClassifier.

    :   **Specified by:**: `getOptions` in interface `OptionHandler`
    :   **Returns:**: an array of strings suitable for passing to setOptions

---


### doTests

```
public abstract void doTests()
```

:   Begin the tests, reporting results to System.out

---


### setDebug

```
public void setDebug(boolean debug)
```

:   Set debugging mode

    :   **Parameters:**: `debug` - true if debug output should be printed

---


### getDebug

```
public boolean getDebug()
```

:   Get whether debugging is turned on

    :   **Returns:**: true if debugging output is on

---


### setSilent

```
public void setSilent(boolean value)
```

:   Set slient mode, i.e., no output at all to stdout

    :   **Parameters:**: `value` - whether silent mode is active or not

---


### getSilent

```
public boolean getSilent()
```

:   Get whether silent mode is turned on

    :   **Returns:**: true if silent mode is on


---


|  |  |  |
| --- | --- | --- |
| **Overview** \| **Package** \| **Class** \| **Tree** \| **Deprecated** \| **Index** \| **Help** \| **Weka's home** | |  |
| **PREV CLASS**   **NEXT CLASS** | **FRAMES**    **NO FRAMES**     **All Classes** |  |
| SUMMARY: NESTED \| FIELD \| CONSTR \| METHOD | DETAIL: FIELD \| CONSTR \| METHOD |  |


---
